# Vascular and immune epigenetic mechanisms underlying Alzheimer's disease

**DOI:** 10.1002/alz70855_102862

**Published:** 2025-12-23

**Authors:** Alexi Nott

**Affiliations:** ^1^ UK Dementia Research Institute, London, United Kingdom; Imperial College London, London, United Kingdom

## Abstract

**Background:**

Alzheimer's disease (AD) is often co‐morbid with cerebral small vessel disease (SVD), and vascular brain endothelial cells (BECs) exhibit a strong expression of genes linked to AD genetic risk. However, the gene regulatory networks of neurovascular cells and their connection to genetic disease risk remain largely unstudied.

**Method:**

To examine noncoding genetic risk variants, we generated enhancer maps for human BECs, mural cells, and other brain cell types using H3K27ac and H3K4me3 CUT&Tag of cell type‐enriched nuclei populations isolated by fluorescence‐activated nuclei sorting (FANS). Enhancers were linked to targets genes using chromatin conformation data we generated for human BECs and mural cells using promoter capture HiC and publicly available data for other brain cell types.

**Result:**

Our enhancer maps for human BECs, mural cells, and other brain cell types revealed AD heritability is primarily related to immune pathways, with moderate enrichment in BECs. In contrast, SVD heritability is broadly enriched across the neurovascular unit, including astrocytes. Enhancer‐to‐gene interactions implicate amyloid‐related processes in both AD and SVD, and gene prioritization and drug analysis point to hypertension medications as potential treatments for AD.

**Conclusion:**

This study highlights the distinct gene regulatory landscapes of neurovascular cells, revealing immune pathway involvement in AD heritability, broad neurovascular enrichment in SVD, with potential therapeutic implications these conditions.

